# Short overview on the current treatment of chronic myeloid leukemia in chronic phase

**DOI:** 10.1007/s12254-016-0299-8

**Published:** 2016-12-14

**Authors:** Stefan Schmidt

**Affiliations:** Universitätsklinik für Innere Medizin V, Hämatologie und Onkologie, Medizinische Universität Innsbruck, Anichstr. 35, 6020 Innsbruck, Austria

**Keywords:** CML, Tyrosine kinase inhibitor, Treatment free remission, Adverse events, ELN recommendations

## Abstract

This short review on current treatment options in chronic myeloid leukemia (CML) in the chronic phase summarizes the latest version of the ELN treatment recommendations dating from 2013 and indicates treatment situations not yet reflected in these recommendations. Daily practice in CML management is complicated by the recently observed treatment-emergent vascular and pulmonary adverse events in second- or later-generation tyrosine kinase inhibitors (TKIs), the lack of guidance with respect to the best TKI for initial treatment, as well as the optimal TKI sequence because no prospective randomized comparative data for second- and third-generation TKIs are available. Physicians have to balance the efficacy issues and safety aspects of the respective TKI and consider patient-specific factors such as comorbidities. Patients with any cardiovascular or pulmonary disease or treatment-requiring cardiovascular risk factor should receive nilotinib or ponatinib only if risk factors and comorbidities are treated accordingly and are further monitored. If these comorbidities are insufficiently controlled, other TKIs might be preferred. Dasatinib treatment should be critically evaluated in patients with pulmonary disease and other TKIs might be preferred in this setting. For as long as CML treatment is considered to be maintained lifelong, and no survival benefit for later-generation TKIs has been demonstrated, safety issues dominate the choice of treatment options. The concept of discontinuing TKI treatment after achieving a deep molecular response might in future change these considerations.

The introduction of tyrosine kinase inhibitors (TKIs) for the treatment of chronic myeloid leukemia (CML) led to a near-normal life expectancy of patients. Alongside this therapeutic success, minimal residual disease quantification by BCR-ABL1 monitoring was shown to be predictive of survival and thus molecular remission became the cornerstone of the treatment goals as recommended by the ELN (see Table 1) and National Comprehensive Cancer Network (NCCN).

**Table 1:** ELN 2013 treatment recommendations

Time	Optimal	Warning	Failure
Baseline	–	High-risk major route CCA/Ph+	–
3 months	BCR-ABLIS < 10%* Ph+ < 35% (PCyR)	BCR-ABLIS > 10%* Ph+ 36–95%	NoCHR* Ph+ > 95%
6 months	BCR-ABLIS < 1%* Ph + 0% (CCyR)	BCR-ABLIS 1–10%* Ph + 1–35%	BCR-ABLIS > 10%* Ph+ > 35%
12 months	BCR-ABLIS < 0.1%* (MMR)	BCR-ABLIS 0.1–1%*	BCR-ABLIS > 1%* Ph+ > 0%
>12 months	MMR or better	CCA/Ph-(-7, or7q-)	Loss of CHR Loss of CCyR Loss of MMR, confirmed** Mutations CCA/Ph+
At 1st-line failure	–	NoCHR Loss of CHR on imatinib Lack of CyRto 1st-line TKI high-risk	–
3 months of 2nd-line	BCR-ABLIS < 10%* Ph+ < 65%	BCR-ABLIS > 10%* Ph+ 65–95%	NoCHR, or Ph+ > 95%, or New mutations
6 months of 2nd-line	BCR-ABLIS < 10%* Ph+ < 35% (PCyR)	BCR-ABLIS < 10%* Ph+ 35–65%	BCR-ABLIS > 10%* Ph+ > 65%* New mutations
12 months of 2nd-line	BCR-ABLIS < 1%* Ph+0 (CCyR)	BCR-ABLIS 1–10%* Ph+ 1–35%	BCR-ABLIS > 10%* Ph+ > 35%* New mutations
>12 months of 2nd-line	MMR or better	CCA/Ph-(-7or7q-) or BCR-ABLIS > 0.1%	Loss of CHR, or Loss of CCyR or PCyR New mutations Loss of MMR** CCA/Ph+

*and/or

**in 2 consecutive tests, of which one ≥1%

Currently five different TKIs are approved for CML treatment. Imatinib and the two second-generation TKIs dasatinib and nilotinib are recommended and approved for first-line treatment. In the case of intolerance, the use of any other TKI approved for first-line therapy is recommended. Patients in whom treatment has failed can in principle receive any other TKI than imatinib. However, according to the European Medicines Agency (EMA) indications, patients either must have failed a prior treatment with dasatinib or nilotinib (ponatinib), or a second-line treatment with imatinib, dasatinib, or nilotinib would have to be unsuitable (bosutinib). An exception is the occurrence of a T315I-mutation of BCR-ABL1, against which no other TKI than ponatinib has shown activity [1].

## First-line treatment

In most cases physicians have to choose between imatinib, dasatinib, and nilotinib for first-line treatment. However, which of these is the best to start with in order to achieve the defined treatment goals remains an unsolved issue. Owing to the lack of randomized direct comparison of dasatinib and nilotinib the choice of the initial TKI is based on balancing the risk of progression against safety issues of the specific TKI in consideration. In the case of high-risk patients according to SOKAL, HASFORD, or EUTOS score, second-generation TKIs (2G-TKIs) are often preferred [2–4], although a newer meta-analysis does not seem to support this approach [5].

Both, the ENESTnd [6] and the DASION [7] trial have shown higher efficacy of the respective 2G-TKIs (nilotinib and dasatinib) when compared with imatinib. Based on data on these and other trials, major molecular response (MMR) rates at 12 months can be expected to be 46% and 51% (vs. 28% and 27% for imatinib) and deep molecular remissions at 5‑year follow-up were observed in 42% and 54% of patients for dasatinib and nilotinib (vs 3% and 1% for imatinib), respectively.

However, the estimated overall 5‑year survival was not significantly different from that of the corresponding imatinib-treated groups (91% and 92.2% for dasatinib and nilotinib, respectively, compared with 90% and 91%). In addition, neither the greater percentage of patients who achieved early molecular response (EMR: <10% BCR-ABL1^IS^ at 3 months of treatment) under 2G-TKI treatment [8, 9] nor the lower rate of progressions into an accelerated or blastic phase observed under this treatment has translated into an overall survival benefit.

Therefore, standard use of 2G-TKIs instead of imatinib in first-line treatment is controversial and patients might be overtreated when receiving a 2G-TKI. Provided that TKI treatment in CML has to be lifelong, safety issues have to take precedence over aspects of efficacy as long as survival is not affected.

Some adverse events impact on the choice of the TKI treatment in particular as they, although rare, result in severe and irreversible morbidity in a certain group of patients. Such events are arterial cardio- and cerebrovascular thromboembolic events as well as occlusive events of the peripheral arteries, which were observed under both nilotinib and ponatinib treatment, venous thrombotic events reported under ponatinib, and the occurrence of pleural effusions and pulmonary arterial hypertension that is associated with dasatinib treatment. Since the presence of cardiovascular risk factors increases the risk of arterial occlusive events occurring under nilotinib and ponatinib treatment [10, 11], these should be corrected. However, therapy with either nilotinib or ponatinib per se in the absence of any other indication for treating these risk factors is no reason to start such treatment [12]. In addition, there are no data on whether such a preemptive treatment can reduce vascular occlusive events.

For low-risk patients, therefore, treatment with imatinib appears to be reasonable. Similarly, in high-risk patients with ongoing and uncontrolled cardiovascular comorbidities, imatinib or dasatinib treatment might be preferred to nilotinib (or in later treatment lines, ponatinib). By contrast, high-risk patients with cardiovascular risk factors or controlled cardiovascular comorbidities might receive a later-generation TKI provided that treatment of the cardiovascular risk factors is continued and a close monitoring of the cardiovascular and metabolic state is available. Such monitoring should include at least a 3‑ to 6‑monthly measurement of ankle–brachial index and of the HbA1C. For high-risk patients with pulmonary comorbidities, other 2G-TKIs than dasatinib should be preferred.

## Adverse events

In general TKIs have a good safety profile; nevertheless, serious adverse events (AEs) occur and even fatal events have been described. Since the latter are so rare and the former are often of mild severity, physicians’ alertness for AEs in TKI treatment may have dwindled. The ELN established a set of recommendations for AE management [12] and the general approach is summarized in Table 2, but more details are beyond the scope of this introduction. Experience in AE management is essential to help overcome any reluctance in re-starting and re-escalating the TKI dose once an AE has occurred. A recent trial on optimizing nilotinib treatment demonstrated that in 26.4% of patients who had their dose previously reduced, no attempt to re-escalate the dose was undertaken. In this group only 57.9% achieved an MMR at 24 months while 84.8% of patients in whom re-escalation was tolerated achieved this response level [13]. Dose re-escalation might be of particular importance in patients in whom cytopenias triggered a dose reduction, because myelosuppression in TKI treatment represents a slow recovery of normal stem cell function from leukemia-induced suppression rather than a true toxic effect of TKIs. Newly developing or progression of previously controlled cardiovascular or pulmonary diseases under nilotinib or ponatinib TKI treatment requires a change of the TKI used.

**Table 2:** Management of adverse events (AE)

General approach	
Grade	Recommendation
Grade 1	No change in TKI treatment, specific AE treatment
Grade 2	Withholding TKI until AE resolves to <grade 2, restart at same dose if first episode otherwise restart TKI at reduced dose. Initial continuation of TKI may be possible under specific AE treatment; however, if the AE does not resolve the TKI should be withheld
Grade 3	Withholding TKI until AE resolves to grade of at least <grade 3 and restart TKI at next lower dose level or withhold TKI until AE <grade 2 but then restart at same dose level. If AE duration ≥4 weeks, discontinue and switch TKI, similarly switch in the case of third episode of same AE
Grade 4	Discontinue current TKI and switch to another TKI

## Second- and later-line treatments

Intolerance of or resistance to a specific TKI usually requires a switch to another treatment line. At the 5‑year update for the DASISION and ENESTnd trials, 39% and 40.1% of patients were no longer still on dasatinib or nilotinib treatment (vs. 37% and 50.2% in the respective Imatinib groups). If patients do not achieve a treatment milestone or lose a previously attained response, the underlying reason has to be determined to allow for an informed choice of the next TKI. The lifelong treatment concept and the mainly asymptomatic disease course prior to treatment start can impact on treatment adherence. The latter is a major factor of therapeutic success, as was shown by several groups [14–17]. While measurement of TKI serum levels – if available – might help to identify the problem, confronting the patient with the results might compromise the physician–patient relationship, which as in any long-term treatment is of particular importance to establish and maintain treatment adherence.

Resistance to TKI treatment occurs in 20–30% of patients in the chronic phase of disease and is in up to 50% of cases mediated by BCR-ABL1 mutations [13]. In vitro sensitivity testing of the more than 100 known BCR-ABL1 mutations [3, 18, 19] to different TKIs can be informative, but the results do not in all cases reflect clinical experience. In Table 3 the clinically most important mutations are listed (reviewed in [3, 18, 19]).

**Table 3:** Clinically relevant BCR-ABL1 mutations

Mutation	Imatinib	Dasatinib	Nilotinib	Bosutinib	Ponatinib
G250E	Efficacy ↓	–	–	Efficacy ↓	–
**Q252H**	Efficacy ↓	Efficacy ↓	–	–	–
**Y253F/H**	Efficacy ↓	–	Efficacy ↓	–	–
**E255K/V**	Efficacy ↓	Efficacy ↓	Efficacy ↓	Efficacy ↓	Efficacy ↓
**T315I**	Ineffective	Ineffective	Ineffective	Ineffective	–
**F317L/V/I/C**	Efficacy ↓	Efficacy ↓	–	–	–
F355V	–	–	Efficacy ↓	–	–
H396R	Efficacy ↓	–	–	–	Efficacy ↓
**V299L**	Efficacy ↓	Efficacy ↓	–	Efficacy ↓	–
**T315A**	–	Efficacy ↓	–	–	–
**F359C/I/C**	Efficacy ↓	Efficacy ↓	–	–	–

In bold print mutations assumed to be clinically relevant in most cited references

↓ means decreased

In general, second-line TKI treatment is based on the BCR-ABL1 mutation status where applicable and otherwise on the TKI safety profile and patient-specific factors (see previous section).

In terms of efficiency, complete cytogenetical response (CCyR) rates were 44–53% for dasatinib and 31–45% for nilotinib, while MMR rates of 29–43% and 28% are reported for dasatinib and nilotinib, respectively [20–22]. Bosutinib after imatinib failure demonstrated CCyR rates of 41–48% [20, 23] and MMR rates of 64%. Ponatinib data refer [24] to a highly pretreated population with 58% patients having received three and more TKIs prior to this ponatinib. In this setting, CCyR and MMR rates were 53% and 39%, respectively. Overall survival rates were reported to be 70–74% and 78% at the 6‑year follow-up for dasatinib and nilotinib, respectively. With a much shorter follow-up of 2 years, the overall survival was 92% for bosutinib and 86% for ponatinib.

**Fig. 1:**
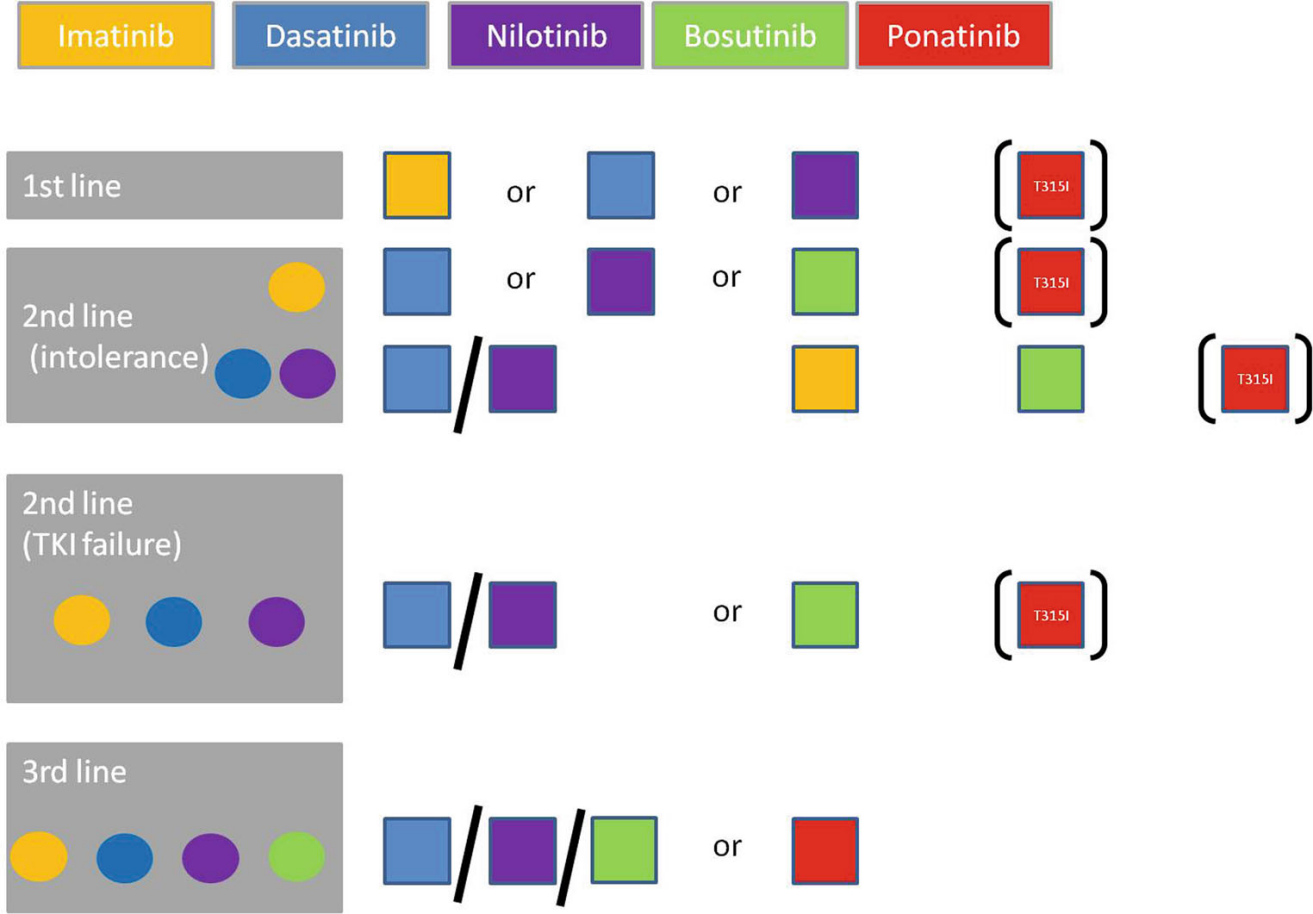
TKI Treatment choices

There are no recommendations for TKI choice in third- or later-line treatment (Fig. 1) due to the few clinical trials in these settings. However, the efficacy of bosutinib, which shares its dual SRC/ABL-inhibitory activity with dasatinib, is markedly reduced (CCyR 17% vs. 54% and MCyR 43% vs. 78%) after imatinib and dasatinib failure compared with its use in dasatinib-naïve patients, e. g., those having failed imatinib and nilotinib [25, 26].

## Beyond standard treatment

There are several new or nonstandard treatment approaches worth mentioning:

- ABL001 is a new class of TKI that is not yet approved for CML treatment [27].
- The efficacy of TKI–interferon combinations [28] is superior to TKI monotherapy.
- High-dose imatinib is superior to standard dose in first-line therapy [29] and optimization of the imatinib dose according to plasma levels markedly increased MMR rates (76% vs. 46–56%) [30]. This might impact treatment decisions in particular once generic imatinib is available.
- Provided the maturing body of evidence supporting its, feasibility treatment-free remission [31] might become part of the standard treatment options and therefore it is noteworthy that more patients under 2G-TKIs than under imatinib 400mg QD achieved the required deep molecular responses.
